# Morbidity and mortality following hiatal hernia repair in geriatric patients: a multicenter research network study

**DOI:** 10.1007/s00464-024-10956-y

**Published:** 2024-06-10

**Authors:** Sunjay S. Kumar, Martina Rama, Scott Koeneman, Sami Tannouri, Talar Tatarian, Francesco Palazzo

**Affiliations:** 1 Department of Surgery, Thomas Jefferson University Hospital, Philadelphia, PA 19107 USA; 2 Sidney Kimmel Medical College, Thomas Jefferson University, 1100 Walnut Street, 5th floor, Philadelphia, PA 19107 USA

**Keywords:** Hiatal, Paraesophageal, Geriatric, Frailty, Asymptomatic

## Abstract

**Background:**

Hiatal hernia is a common surgical pathology. Such hernias can be found incidentally and patients may opt for an initial nonoperative approach though many will pursue surgery after symptom progression. Data on the effects of age on the outcomes of hiatal hernia repair may help inform this decision-making process.

**Methods:**

The TriNetX database was queried for all adult patients undergoing hiatal hernia repair from 2000 to 2023. Patients were divided into elective and emergent cohorts on the basis of diagnosis codes indicating obstruction or gangrene. Patients aged 80–89 were compared against those aged 65–79 in unadjusted analysis. Logistic regression models controlling for additional health history covariates were created to calculate odds ratios for primary outcomes.

**Results:**

There were 2310 octogenarians and 15,295 seniors who underwent elective hiatal hernia repair, and 406 octogenarians and 1462 seniors who underwent emergent repair during the study period. The vast majority of patients in both groups underwent minimally invasive operations. In the elective cohort, octogenarians had higher rates of mortality, malnutrition, sepsis, respiratory failure, pneumonia, DVT, blood transfusion, and discharge to nursing facility. In the emergent cohort, octogenarians had higher rates of mortality, malnutrition, sepsis, and respiratory failure. The odds ratios for mortality in the elective and emergent cohorts were 3.9 (95% CI 3.1–5.0) and 3.5 (95% CI 2.1–5.6), respectively.

**Conclusion:**

Octogenarians are at a meaningfully increased risk for mortality and morbidity after both elective and emergent hiatal hernia repair compared to senior-aged patients. Greater consideration should be given to surgical repair prior to the 8th decade of life.

**Supplementary Information:**

The online version contains supplementary material available at 10.1007/s00464-024-10956-y.

Hiatal hernia is a common surgical pathology, with over 60,000 repairs performed in the United States in 2018 [1]. Many such hernias are found incidentally, leaving the surgeon and patient with a difficult management decision [2]. Given the association between age and size of hiatal hernia, this will become a more frequent dilemma as the population continues to age [3]. Data on the effects of age on the outcomes of hiatal hernia repair may help inform the decision-making process.

The management of hiatal hernia has undergone many iterations over the last century. In 1919, Dr. Soresi published the first account of elective repair of hiatal hernias and advocated for the routine repair of even very small hernias [4]. Fifty years later, some surgeons advocated for observation of minimally symptomatic hernias while others, including Dr. Belsey, advocated for the repair of every paraesophageal hernia [5, 6]. This recommendation was largely born out of concern regarding the risk of gastric volvulus with strangulation.

In 2013, the Society of American Gastrointestinal and Endoscopic Surgeons (SAGES) published its Guidelines for the Management of Hiatal Hernia [7]. One of its recommendations was that “Routine elective repair of completely asymptomatic paraesophageal hernias may not always be indicated.” This recommendation was made on the basis of a Markov Monte Carlo decision analytic model by Stylopoulos et al. that found the average 65 year-old patient with a minimally symptomatic paraesophageal hernia would benefit more from a strategy of watchful waiting than operative management [8].

This model assumed 13.87% of the observation population would crossover into surgical management due to symptom progression each year. However, it did not account for worse outcomes in this scenario due to elective repair at an older age. Additionally, outcomes of paraesophageal hernia repair have improved as cases centralize to high volume centers and care improves [9].

This study was undertaken to delineate the modern outcomes of hiatal hernia repair in an elderly patient cohort compared to a younger cohort to help describe the potential consequences of delaying elective hiatal hernia repair until later in life.

## Material and methods

This population-based, retrospective cohort study was conducted using TriNetX (Cambridge, Massachusetts), a federated health network research database. TriNetX is a multicenter electronic health research network that provides de-identified electronic health record (EHR) data from participating health care organizations (HCOs). Clinical variables are derived directly through the EHRs. Within TriNetX, we utilized data extracted from the Research Network, which is composed of 68 HCOs. ICD-9, ICD-10, and CPT codes were used to extrapolate diagnostic and procedural information. Details of codes utilized for patient selection are described in Supplemental Material 1.

**Fig. 1:**
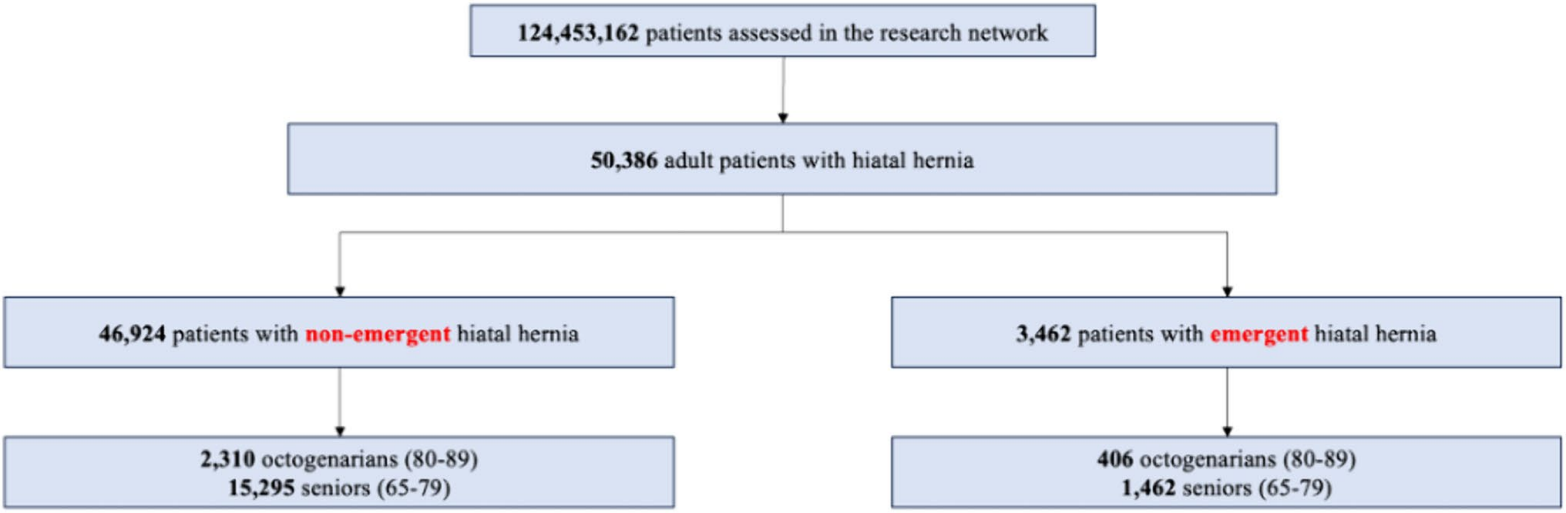
Flowchart for selection of patients who underwent non-emergent or emergent hiatal hernia repair

All adult patients (age ≥ 18 years) with a diagnosis of hiatal hernia from 2000 to 2023 in the US were included in this study. Two separate cohorts were created: an emergent hiatal hernia repair cohort and an elective cohort. The emergent cohort was created by selecting patients with a diagnosis code indicating obstruction or gangrene and hiatal hernia operation within 3 days of diagnosis. The elective cohort was created by selecting patients with a diagnosis of hiatal hernia without obstruction or gangrene and hiatal hernia operation at any time after diagnosis. The hiatal hernia repair is considered the index event in this analysis. Within each cohort, two separate groups were created based on age at index operation: the senior group, which included patients aged 65–79 years, and the octogenarian group, which included patients aged 80–89 years (Fig. 1).

The primary outcomes of this study were mortality, acute respiratory failure, and sepsis. The secondary outcomes included reoperation, malnutrition, pneumonia, deep vein thrombosis (DVT), pulmonary embolism (PE), blood transfusion, need for physical therapy, and discharge to a nursing facility. All outcomes reported in this manuscript were measured at 90 days.

All statistical analysis was performed in R software version 4.3.1 with data obtained from the TriNetX platform. For all primary and secondary outcomes, the absolute risk difference (ARD) between the octogenarian group and the senior group was calculated for the elective and emergent cohorts separately. Additionally, 95% confidence intervals (CI) for these ARDs were calculated. For each primary outcome in each cohort, a logistic regression model was generated, modeling the log odds of the outcome of interest as a function of the following covariates: octogenarian status, history of obesity, history of ischemic heart disease, history of chronic lower respiratory disease, and history of tobacco use. The additional health history covariates were chosen to control for their effects. Using these logistic regression models, the odds ratio (OR) for octogenarians as compared to seniors was estimated for each primary outcome, with accompanying Wald Cis and *p*-values to test the null hypothesis that the odds of each outcome was no different between octogenarians and seniors. To control for multiplicity, we employed a Bonferroni correction across our six comparisons in order to fix our family-wise error rate to be 0.05, and thus we performed each individual test at the 0.00625 level.

This study received a waiver of review from the institutional review board as the TriNetX database includes only aggregated counts and statistical summaries of de-identified information.

## Results

A total of 124,453,162 patients were assessed in the Research Network. Of these, 46,924 were adults who underwent elective hiatal hernia repair, and 3462 were adults who underwent emergent hiatal hernia repair. In the non-emergent cohort, 2310 patients (13.2%) were octogenarians and 15,295 patients (86.8%) were seniors; in the emergent cohort, 406 patients (21.7%) were octogenarians and 1462 patients (78.3%) were seniors. Patient characteristics of the two cohorts are presented in Table 1. With the exception of age, the two cohorts were quite similar. Predictably, octogenarians in both the elective and emergent groups more frequently had hypertensive disease, ischemic heart disease, cerebrovascular disease, and failure to thrive. They had lower rates of nicotine or tobacco use than seniors. Within the elective cohort, octogenarians had a lower rate of obesity. TriNetX does not provide BMI data.

**Table 1:** Baseline characteristics of octogenarians (80–89) and seniors (65–79) in the non-emergent and emergent hiatal hernia repair cohorts

	Elective HH repair		Emergent HH repair	
	*n* (%, 95% CI)		*n* (%, 95% CI)	
	Octogenarian	Senior	Octogenarian	Senior
	*n* = 2310	*n* = 15,295	*n* = 406	*n* = 1462
Age, mean	82.6	72.6	83	71.9
Females	1539 (66.6%)	10,692 (69.9%)	241 (59.4%)	963 (65.9%)
White	1910 (82.7%)	12,634 (82.6%)	326 (80.3%)	1188 (81.3%)
African American	50 (2.2%)	569 (3.7%)	6 (1.5%)	41 (2.8%)
Other	58 (2.5%)	407 (2.7%)	16 (3.9%)	31 (2.1%)
Comorbidities				
Overweight and obese	479 (20.7%)	5048 (33.0%)	71 (17.5%)	434 (20.7%)
Type 2 diabetes	417 (18.1%)	2867 (18.7%)	69 (17.0%)	266 (18.2%)
Hypertensive disease	1800 (77.9%)	10,345 (67.6%	334 (82.3%)	1031 (70.5%)
Ischemic heart disease	796 (34.5%)	3543 (23.1%)	151 (37.2%)	351 (24.0%)
Cerebrovascular disease	414 (17.9%)	1471 (9.6%)	75 (18.5%)	144 (9.9%)
Chronic lower respiratory disease	768 (33.2%)	5014 (32.8%)	129 (31.8%)	465 (31.8%)
Nicotine or tobacco use	98 (4.2%)	1133 (7.4%)	20 (4.9%)	123 (8.4%)
Underweight	9 (0.4%)	37 (0.2%)	4 (1.0%)	5 (0.3%)
Failure to thrive	53 (2.3%)	135 (0.9%)	18 (4.4%)	27 (1.8%)

*HH* hiatal hernia, *CI* confidence interval

The majority of operations were completed with a laparoscopic approach in both the elective and emergent cohorts (Table 2).

**Table 2:** Cases by operative approach within both the elective and emergent cohorts

	Elective HH		Emergent HH	
	*n* (%)		*n* (%)	
	Octogenarian	Senior	Octogenarian	Senior
Open abdominal approach	106 (4.58%)	502 (3.28%)	51 (12.56%)	164 (11.21%)
Open thoracic approach	20 (0.86%)	229 (1.49%)	10 (2.46%)	44 (3.00%)
Thoracoabdominal approach	7 (0.30%)	45 (0.29%)	2 (0.49%)	11 (0.75%)
Laparoscopic approach	2177 (94.24%)	14,519 (94.92%)	343 (84.48%)	1243 (85.02%)
Total	2310	15,295	406	1462

*HH*: hiatal hernia

Within the elective cohort, octogenarians were at increased risk for mortality (ARD 3.6%, 95% CI 2.7–4.5%), malnutrition (ARD 3.6%, 95% CI 2.6–4.7%), sepsis (ARD 1.9%, 95% CI 1.1–2.7%), respiratory failure (ARD 3.3%, 95% CI 2.3–4.2%), pneumonia (ARD 2.8%, 95% CI 1.8–3.8%), DVT (ARD 1.4%, 95% CI 0.6–2.1%), blood transfusion (ARD 0.6%, 95% CI 0.1–1%), and discharge to a nursing facility (ARD 0.9%, 95% CI 0.3–1.5%) (Table 3). Within the emergent cohort, octogenarians were at increased risk for mortality (ARD 6.7%, 95% CI 3.6–9.7%), malnutrition (ARD 6.3%, 95% CI 2.8–9.7%), sepsis (ARD 4.7%, 95% CI 1.7–7.8%), and respiratory failure (ARD 5.9%, 95% CI 2.5–9.2%) (Table 4).

**Table 3:** 90 day outcomes in the elective repair cohort

	Elective HH		
	*n* (%)		
	Octogenarian	Senior	Absolute risk difference (95% CI)
*n*	2310	15,295	
Mortality	109 (4.7%)	169 (1.1%)	3.6% (2.7%, 4.5%)
Reoperation	12 (0.5%)	71 (0.5%)	0.1% (− 0.3%, 0.4%)
Malnutrition	146 (6.3%)	409 (2.7%)	3.6% (2.6%, 4.7%)
Sepsis	78 (3.4%)	223 (1.5%)	1.9% (1.1%, 2.7%)
Respiratory failure	127 (5.5%)	343 (2.2%)	3.3% (2.3%, 4.2%)
Pneumonia	119 (5.2%)	360 (2.4%)	2.8% (1.8%, 3.8%)
DVT	66 (2.9%)	226 (1.5%)	1.4% (0.6%, 2.1%)
PE	41 (1.8%)	261 (1.7%)	0.1% (− 0.5%, 0.7%)
Blood transfusion	27 (1.2%)	93 (0.6%)	0.6% (0.1%, 1%)
Need for Physical therapy	98 (4.2%)	645 (4.2%)	< 0.0% (− 0.9%, 0.9%)
Discharge to nursing facility	41 (1.8%)	129 (0.8%)	0.9% (0.3%, 1.5%)

**Table 4:** 90 day outcomes in the emergent repair cohort

	Emergent HH		
	*n* (%)		
	Octogenarian	Senior	Absolute risk difference (95% CI)
*n*	406	1462	
Mortality	37 (9.1%)	36 (2.5%)	6.7% (3.6%, 9.7%)
Reoperation	2 (0.5%)	11 (0.8%)	− 0.3% (− 1.2%, 0.7%)
Malnutrition	46 (11.3%)	74 (5.1%)	6.3% (2.8%, 9.7%)
Sepsis	35 (8.6%)	57 (3.9%)	4.7% (1.7%, 7.8%)
Respiratory failure	43 (10.6%)	69 (4.7%)	5.9% (2.5%, 9.2%)
Pneumonia	29 (7.1%)	73 (5.0%)	2.1% (− 0.8%, 5.1%)
DVT	18 (4.4%)	44 (3.0%)	1.4% (− 0.9%, 3.8%)
PE	10 (2.5%)	39 (2.7%)	− 0.2% (− 2.0%, 1.7%)
Blood transfusion	6 (1.5%)	19 (1.3%)	0.2% (− 1.3%, 1.6%)
Need for Physical therapy	22 (5.5%)	77 (5.3%)	0.2% (− 2.5%, 2.8%)
Discharge to nursing facility	11 (2.7%)	34 (2.3%)	0.4% (− 1.5%, 2.3%)

**Fig. 2:**
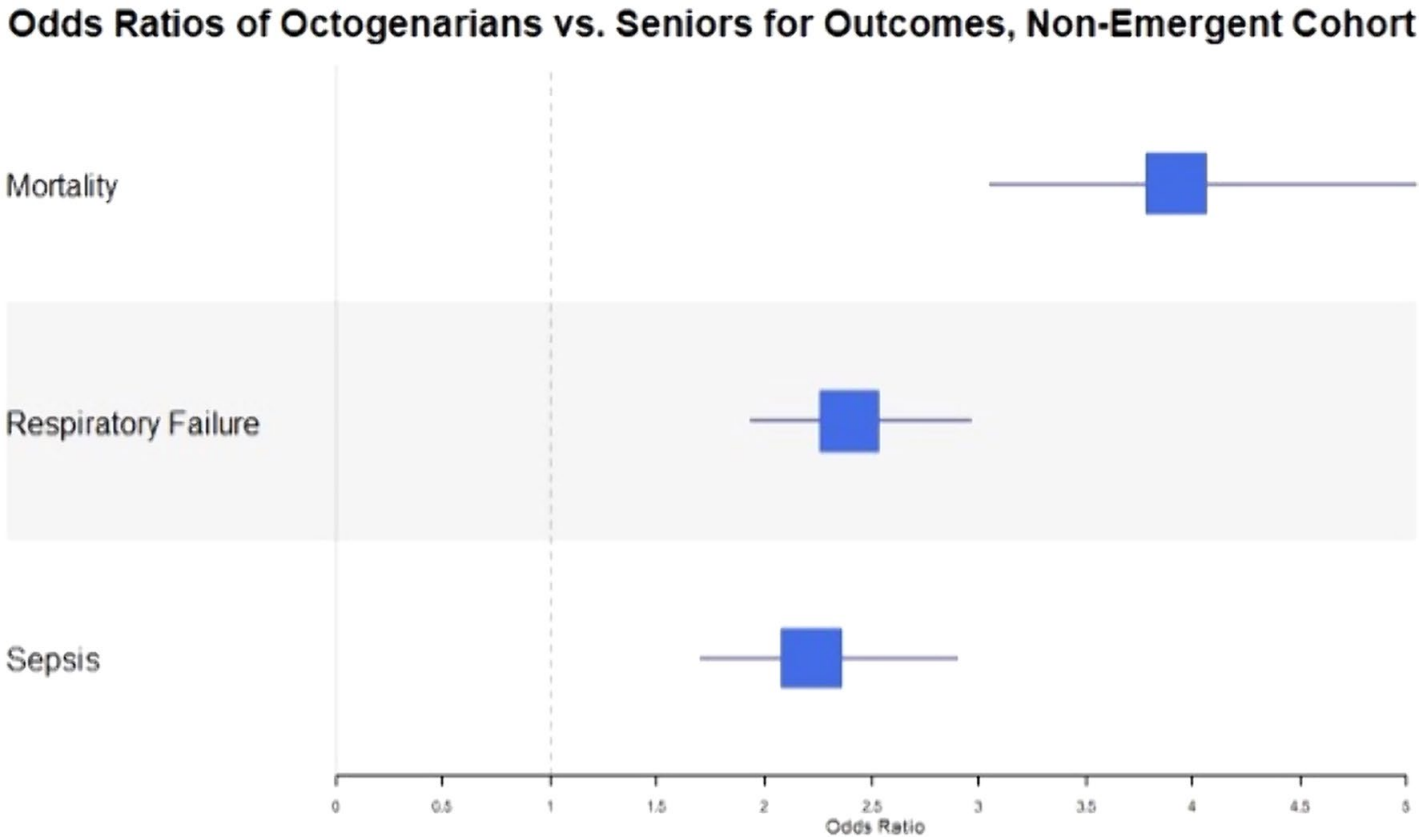
Odds ratios of octogenarians vs seniors for primary outcomes in the elective cohort

**Fig. 3:**
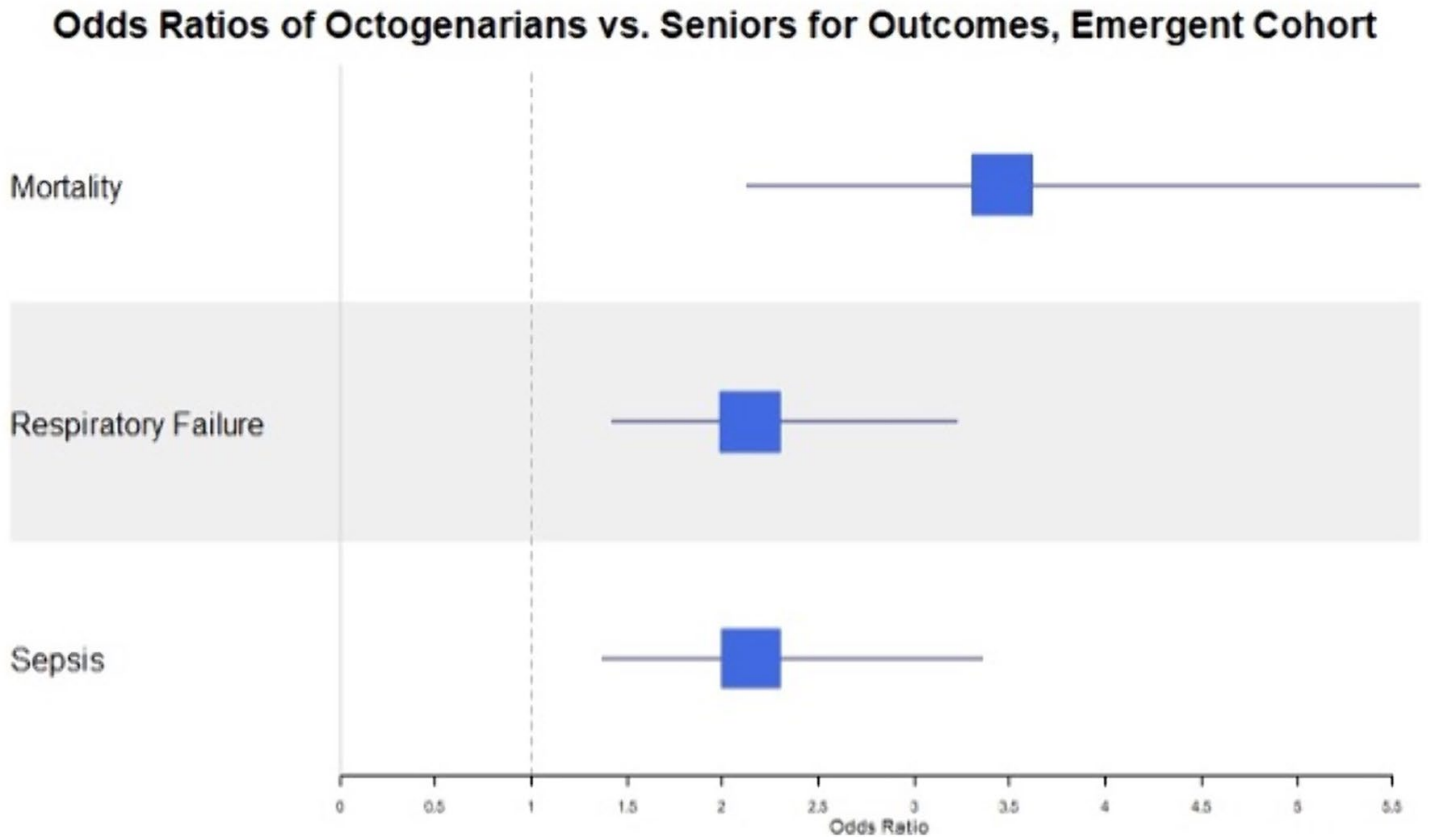
Odds ratios of octogenarians versus seniors for primary outcomes in the emergent cohort

Utilizing the logistic regression models, the OR for mortality in octogenarians as compared to seniors was 3.9 (95% CI 3.1–5.0) in elective cases and 3.5 (95% CI 2.1–5.6) in emergent cases. The OR for respiratory failure was 2.4 (95% CI 1.9–3.0) for elective cases and 2.1 (95% CI 1.4–3.2) for emergent cases. The OR for sepsis was 2.2 (95% CI 1.7–2.9) in elective cases and 2.1 (95% CI 1.4–3.4) for emergent cases. These results are presented in Figs. 2, 3.

## Discussion

This retrospective study utilizing the TriNetX database found that octogenarians undergoing both elective and emergent hiatal hernia repair are at significantly increased risk for death and other complications compared to patients aged 65–79. These effects persisted after controlling for meaningful differences in comorbidities between the two groups. This suggests that senior-age patients who delay elective repair of hiatal hernia until the 8th decade of life are exposing themselves to significantly elevated risk for poor outcomes.

Wilson et. al have recently published data with a similar design, using the Nationwide Readmission Database to compare octogenarians against adult patients aged 18–79 undergoing paraesophageal hernia repair [10]. After controlling for multiple variables including Charlson Comorbidity Index scores, they did not find that octogenarian status was interpedently predictive of mortality, though undergoing an emergent operation was. This is somewhat surprising given the much younger cohort they were comparing against octogenarians but may be related to the number of variables controlled for in their models.

Single institution data has previously demonstrated that both age over 72 and high frailty double the odds of morbidity after elective repair of paraesophageal hernia [11]. This study replicates a similar finding on the effects of age in a national database. Age and frailty have similarly been found to be meaningful predictors of mortality after emergent repair in large, national databases [12]. These findings are consistent with the current study, even though frailty was not directly assessed, as the variables controlled for in the multivariate model have some overlap with the modified frailty index.

The Stylopoulos paper has been followed by two similar papers utilizing relatively more modern data [8, 13, 14]. At the time of this manuscript preparation, these three papers are important citations in the proposed update to the SAGES Guideline for Hiatal Hernia Repair which is currently available for public comment [15]. Interestingly, all three of these models assume a mortality of 5.4–5.5% in emergent repairs while the mortality rate across patients age 65–89 in this cohort was 3.9%.

The data on the progression from asymptomatic hiatal hernias to symptomatic or emergent is very limited. In light of the increased utilization of minimally invasive surgery, its increased safety profile, and the reported mortality rates, the Authors would encourage further study. As data accumulates, a reevaluation of recommendations addressing whether to operate on the asymptomatic hiatal hernia in the senior or geriatric population may be warranted. A European expert Delphi consensus published in 2023 sought to answer the question of indications for surgical repair of paraesophageal hernia and may signal a change in surgeons’ perspective [16]. They labeled surgery the “recommended” therapeutic strategy for symptomatic patients regardless of age and considered surgery “acceptable” for patients with no or minor symptoms.

This study has a number of limitations. Type I, or sliding, hiatal hernias were likely included so it is not truly representative of paraesophageal hernias alone. However this is unlikely to minimize the demonstrated effects. This study also had no ability to distinguish the operation each patient received.

Avenues for future research include further differentiating the effects of age versus frailty on the outcomes of elective hiatal hernia repair. The models investigating the outcomes of elective hiatal hernia repair versus observation may also benefit from being updated with more modern data.

The results of this study strongly suggest that senior-age patients considering hiatal hernia repair would have significantly better outcomes with immediate elective repair rather than pursuing repair after age 80.

### Supplementary Information

Below is the link to the electronic supplementary material.

Supplementary file1 (DOCX 15 kb)
