# Supplementary material for: Morbidity and mortality following hiatal hernia repair in geriatric patients: a multicenter research network study
Source: Surg Endosc. 2024 Jun 10;38(7):3999–4005. doi: 10.1007/s00464-024-10956-y (PMC11219371; doi:10.1007/s00464-024-10956-y)
Supplement: Supplementary file 1 — Supplementary file1 (DOCX 15 kb) [file 464_2024_10956_MOESM1_ESM.docx]

Supplement 1

**Selection of patients**

The search was conducted following the criteria provided by TriNetX to identify potential patients. These codes include the International Classification of Diseases, Ninth and Tenth Revisions, and Clinical Modification (ICD-9, ICD-10) and Current Procedural Terminology (CPT) codes.

Diagnosis and procedure codes to assist in identifying emergent hiatal hernia cohort:

All patient with a diagnosis of emergent hiatal hernia and who underwent hiatal hernia operation within 3 days of diagnosis were included in this cohort. Codes:

Emergent hiatal hernia diagnosis:

- K44.0, 552.3: diaphragmatic hernia with obstruction, without gangrene
- K44.1, 551.3: diaphragmatic hernia with gangrene

Repair:

- 43332: repair, paraesophageal hiatal hernia (including fundoplication), via laparotomy, except neonatal; without implantation of mesh or other prosthesis
- 43333: repair, paraesophageal hiatal hernia (including fundoplication), via laparotomy, except neonatal; with implantation of mesh or other prosthesis
- 43334: repair, paraesophageal hiatal hernia (including fundoplication), via thoracotomy, except neonatal; without implantation of mesh or other prosthesis
- 43335: repair, paraesophageal hiatal hernia (including fundoplication), via thoracotomy, except neonatal; with implantation of mesh or other prosthesis
- 43336: repair, paraesophageal hiatal hernia (including fundoplication), via thoracoabdominal incision, except neonatal; without implantation of mesh or other prosthesis
- 43337: repair, paraesophageal hiatal hernia (including fundoplication), via thoracoabdominal incision, except neonatal; with implantation of mesh or other prosthesis
- 43281: laparoscopy, surgical, repair of paraesophageal hernia, includes fundoplasty, when performed; without implantation of mesh
- 43282: laparoscopy, surgical, repair of paraesophageal hernia, includes fundoplasty, when performed; with implantation of mesh

Diagnosis and procedure codes to assist in identifying non-emergent hiatal hernia cohort:

All patient with a diagnosis of non-emergent hiatal hernia and who underwent hiatal hernia operation any time after the diagnosis were included in this cohort. Codes:

Non-emergent hiatal hernia diagnosis:

- K44.9, 553.3: diaphragmatic hernia without obstruction or gangrene

Repair:

- 43332: repair, paraesophageal hiatal hernia (including fundoplication), via laparotomy, except neonatal; without implantation of mesh or other prosthesis
- 43333: repair, paraesophageal hiatal hernia (including fundoplication), via laparotomy, except neonatal; with implantation of mesh or other prosthesis
- 43334: repair, paraesophageal hiatal hernia (including fundoplication), via thoracotomy, except neonatal; without implantation of mesh or other prosthesis
- 43335: repair, paraesophageal hiatal hernia (including fundoplication), via thoracotomy, except neonatal; with implantation of mesh or other prosthesis
- 43336: repair, paraesophageal hiatal hernia (including fundoplication), via thoracoabdominal incision, except neonatal; without implantation of mesh or other prosthesis
- 43337: repair, paraesophageal hiatal hernia (including fundoplication), via thoracoabdominal incision, except neonatal; with implantation of mesh or other prosthesis
- 43281: laparoscopy, surgical, repair of paraesophageal hernia, includes fundoplasty, when performed; without implantation of mesh
- 43282: laparoscopy, surgical, repair of paraesophageal hernia, includes fundoplasty, when performed; with implantation of mesh

Diagnosis and procedure codes to assist in identifying comorbidities:

- E66, 278.0: Overweight and obesity:
- E11, 250.20, 250.22, 250.32, 250.42, 250.40, 250.50, 250.52, 250.60, 250.62, 250.70, 250.72, 250.80, 250.30, 250.02, 250.12, 250.22, 250.32, 250.42, 250.52, 250.62, 250.72, 250.80, 250.82, 250.92, 250.10, 250.12, 250.90, 250.92, 250.00: Type 2 Diabetes Mellitus
- I10, 1I11, I13, I15, I16, I1A, 401, 402: Hypertensive disease
- I20-I25, 413, 410, 429.7, 429.2, 412, 414, 411: Ischemic heart disease
- I60-I69, 430, 431, 432, 433, 434, 437.3, 437, 435, 436, 438.8, 438.9, 438.1, 438: Cerebrovascular disease
- J40, J41, J42, J43, J44, J45, J47, J4A, 490, 491, 492, 493, 496, 494: Chronic lower respiratory disease
- F17, Z72.0, 305.1, 292.8: Nicotine dependence/tobacco use
- R63.6, 783.22: Underweight
- R62.7, 783.7: Failure to thrive

Diagnosis and procedure codes to assist in identifying outcomes of interest:

- E40-E46, 260, 261, 262, 263, 263.1, 263.2, 263.8, 263.9, R63.4, 783.3: Malnutrition
- 43289, 43282: Reoperation
- I82.6, I82.40, I82.41, I82.42, I82.43, I82.44, I82.45, I82.46, I82.49, I82.4Y, I82.4Z, 453.40, 453.41, 453.42, 453.83, 453.81, 453.82: Deep vein thrombosis (DVT)
- I26, 415, 415.11, 415.12, 415.13, 415.1, 415.19: Pulmonary embolism (PE)
- A41, 038: Sepsis
- J96.0, 518.51, 518.81: acute respiratory failure
- J18, 483, 485, 481, 486: pneumonia
- K56.7, K56.6, 560.1, 531.01, 531.11, 531.21, 531.31, 531.41, 531.51, 531.61, 531.71, 531.91, 532.01, 532.11, 532.21, 532.41, 532.51, 532.61, 532.71, 532.91, 560.9, 560, 560.8, 560.89: Ileus/obstruction
- 36430: Blood transfusion
- 1029677, Z51.89, F07, F08, V58, V66: Physical therapy
- Z99.3, V46.3: Wheelchair dependency
- 1013753, 99316, 99315, 1013754: Discharge to nursing facility
- 5A09357, 5A09358, 5A09359, 5A0935B, 5A09457, 5A09458, 5A09459, 5A0945B, 5A09557, 5A09558, 5A09559, 5A0955B, 5A09B5K, 5A09C5K, 5A09D5K, 94002: Mechanical respiratory ventilation
- 5A2204Z, 99.61, 99.62, 99.64, 99.69, 92950: Cardiopulmonary resuscitation (CPR)
